# Variability of Grading DR Screening Images among Non-Trained Retina Specialists

**DOI:** 10.3390/jcm11113125

**Published:** 2022-05-31

**Authors:** Andrzej Grzybowski, Piotr Brona, Tomasz Krzywicki, Magdalena Gaca-Wysocka, Arleta Berlińska, Anna Święch

**Affiliations:** 1 Department of Ophthalmology, University of Warmia and Mazury, 10-719 Olsztyn, Poland; arleta.berlinska@gmail.com; 2 Institute for Research in Ophthalmology, Foundation for Ophthalmology Development, 60-553 Poznan, Poland; 3 Department of Ophthalmology, Poznan City Hospital, Szwajcarska 3, 60-285 Poznan, Poland; piotrbrona@gmail.com (P.B.); mgacawysocka@gmail.com (M.G.-W.); 4 Department of Mathematical Methods of Informatics, University of Warmia and Mazury, 10-719 Olsztyn, Poland; tomasz.krzywicki@matman.uwm.edu.pl; 5 Department of Vitreoretinal Surgery, Medical University of Lublin, 20-093 Lublin, Poland; anna.zub@umlub.pl

**Keywords:** grader comparison, diabetic retinopathy grading, deep learning, inter-grader variability, diabetic retinopathy screening

## Abstract

Poland has never had a widespread diabetic retinopathy (DR) screening program and subsequently has no purpose-trained graders and no established grader training scheme. Herein, we compare the performance and variability of three retinal specialists with no additional DR grading training in assessing images from 335 real-life screening encounters and contrast their performance against IDx-DR, a US Food and Drug Administration (FDA) approved DR screening suite. A total of 1501 fundus images from 670 eyes were assessed by each grader with a final grade on a per-eye level. Unanimous agreement between all graders was achieved for 385 eyes, and 110 patients, out of which 98% had a final grade of no DR. Thirty-six patients had final grades higher than mild DR, out of which only two had no grader disagreements regarding severity. A total of 28 eyes underwent adjudication due to complete grader disagreement. Four patients had discordant grades ranging from no DR to severe DR between the human graders and IDx-DR. Retina specialists achieved kappa scores of 0.52, 0.78, and 0.61. Retina specialists had relatively high grader variability and only a modest concordance with IDx-DR results. Focused training and verification are recommended for any potential DR graders before assessing DR screening images.

## 1. Introduction

Automatic analysis of image studies, particularly fundus photographs with the use of machine learning and other similar “artificial intelligence” (AI) applications, is a rapidly developing field. Diabetic retinopathy (DR) is one of the leading causes of vision loss worldwide; as the prevalence of diabetes increases, so does the burden on health services. Automated screening software is one of the proposed solutions to this issue. Main benefits for regions with established DR screening schemes is a reduction in costs associated with manual grading. At the same time, with their introduction, it may become feasible to establish new DR screening initiatives in poor, health-service-deprived, and remote regions.

DR grading based on fundus images is a multi-step process that requires identification and differentiation of a number of key features, such as retinal hemorrhages, microaneurysms, hard exudates, new vessels, intraretinal microvascular abnormalities, neovascularization, and markers of macular edema. This is further complicated by variations of image quality, brightness, and the presence of retinal changes unassociated with DR. On the whole, this leads to a fair amount of both inter-grader and intra-grader variability [1]. This is compatible with grader variability seen in other fields of medical imaging [1,2,3,4].

Firstly, commonly accepted criteria for grading DR were established by Airlie House classifications and later refined and expanded by the Early Treatment of Diabetic Retinopathy Study (ETDRS) [5]. The ETDRS grading scale was developed in order to standardize and quantitatively stratify the results of this and other investigations into DR and its treatment. The grading scheme is based on standardized seven-field fundus photography, and grading is based on a comparison against standardized photos of microaneurysms, hemorrhages, and new vessels [6]. Overall, the original ETDRS criteria are rather convoluted, with each retinal feature having its own grading scheme set against the standard image comparison. The International Clinic Diabetic Retinopathy (ICDR) scale was first published in 2003 and was developed by a working group of 31 retina specialists, epidemiologists, and endocrinologists. This classification was created to simplify the ETDRS scale and create a grading system that would be viable in a daily practice and smaller study settings [7].

AI algorithms rely heavily on a human-grader-based reference standard on all stages of development. Deep learning is a way of teaching computer software the most predictive features of an image without designing specific rules or features to be analyzed. The primary requirement for developing a deep learning algorithm is a sufficiently large base of images with labeled outcomes. The software “learns” what constitutes a certain grade of DR or referable DR based on those labels. Development of an accurate DR screening system therefore relies on accurate interpretation of the images by human graders beforehand. After an algorithm is established, it is verified against human grader decisions. To strengthen the human-grader-based standard, multiple graders are sometimes involved in reaching a final decision.

Poland has never had a widespread traditional or fundus-image-based DR screening program. As a result, there are no trained and experienced DR graders, no established training scheme for graders, and a lack of trained ophthalmic imaging technicians. We decided to look into the grading performance and variability of retina specialists in Poland and contrast their performance with a recognized, FDA-approved DR screening suite—IDX-DR.

## 2. Methodology

We sourced images from a local AI-based DR screening program. This is a DR screening program based around diabetology clinics offering diabetics free DR checkups using full size fundus cameras and IDx-DR AI-medical device (Digital Diagnostics Inc., Oakdale, IN, USA). We selected patients that visited DR screening site between January 2020 and June 2021 for a total of 495 patients. All images were taken using Topcon NW-400 automatic fundus camera by local staff after training. We collected fundus images taken at the testing site and sent them to three separate human graders for DR grading.

The three human graders were all consultant ophthalmologists with particular interest and long-term work in retinology. This is what we consider to be an equivalent of being a retina specialist in Poland, as no officially sanctioned subspecialty distinctions exist. Because DR screening has never been organized on a national level in Poland and regional programs are sparse, none of the graders had prior extensive experience of grading DR from fundus images. The graders reviewed individual eyes according to ICDR criteria, in a 5-point scale. Surrogate markers of diabetic macular edema were not tracked separately but were taken into account by reviewers when designating final grading for individual eyes. In addition, we assigned each patient an automatic diagnosis obtained using the IDx-DR (in a 4-point-scale). The image sets for patients contained a variable number of images per eye, often with mixed quality of the images. Patients for which at least one grader designated an insufficient quality marking in at least one eye were excluded from further analysis.

In the next step, we prepared two sets of ratings: (1) containing one DR diagnosis assigned by the human graders in each eye for each patient; (2) containing the highest DR diagnosis out of both eyes to reach a patient-level human grader diagnosis. In addition, we assigned each patient a diagnosis using IDx-DR software. We scaled all the DR grades assigned by the human graders in the second set of ratings to a 4-point scale. To match the grading output of IDx-DR, we scaled the two highest DR results—severe proliferative and severe non-proliferative diabetic retinopathy—achieving a 4-point scale.

In cases with discordant rating of an eye between the graders, in order to determine a final gold standard, a majority decision of 2 out of 3 ophthalmologists was chosen. In cases where all three graders designated a different DR grade to an eye, an adjudication process was undertaken. The adjudication process consisted of a remote teleconference meeting where the graders discussed individual eyes and their results until a consensus was reached.

Next, we determined the agreement between the grader diagnoses collected in both sets of ratings. We calculated the number of concordant grader ratings in the following cases: (1) both eyes in all patients; (2) left and right eye separately for all patients; (3) the highest assigned grade assigned by each grader in each patient. In each of the three cases, we determined the number of concordant grader ratings according to the following proportions:

- 1:1:1 (complete incompatibility between the three human graders);
- 2:1 (agreement between two of three human graders);
- 3:0 (complete agreement between three human graders);
- 1:1:1:1 (complete incompatibility between the three graders, and IDx-DR);
- 2:1:1 (agreement between two of three graders, IDx-DR result different than all other graders);
- 2:1:1 (agreement between one of three graders and IDx-DR, where the grade assigned by IDx-DR is subsequently in the majority);
- 2:2 (half compatibility between four graders, including IDx-DR);
- 3:1 (complete agreement between three graders, IDx-DR results different than grader agreement);
- 3:1 (agreement between two of three graders, where the grade assigned by IDx-DR is aligned with the majority);
- 4:0 (complete agreement between three graders and IDx-DR).

For each proportion of ratings, we designated a percentage. In addition, we determined the number of patients and images that had each DR grade assigned for each case of complete grader agreement.

## 3. Results

**Figure 1:**
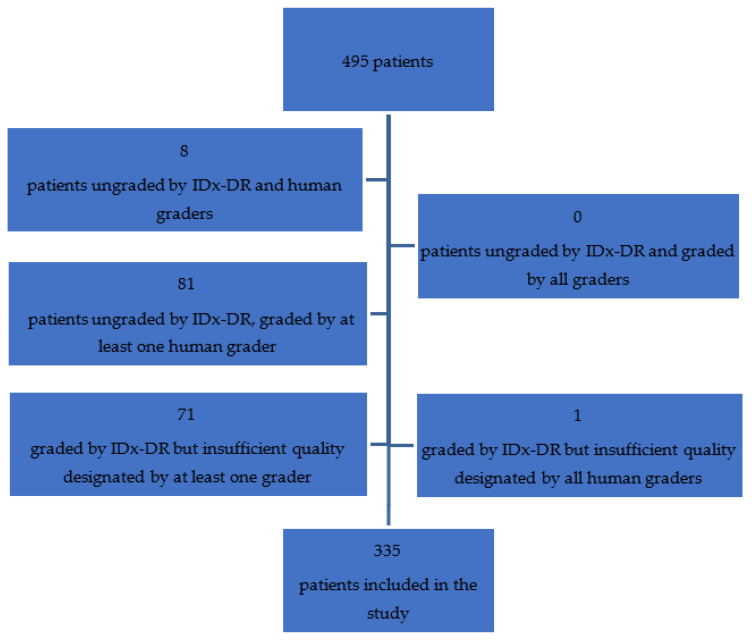
Patients selection flowchart.

In our study, we included 335 thoroughly evaluated patients out of 495 total and 1501 fundus images, as shown in Figure 1. Eight patients had images of insufficient quality for all human graders and IDx-DR to reach a decision. A further 71 patients who have already been graded by IDx-DR were excluded from analysis due to issues surrounding the quality of images raised by at least 1 human grader. A single patient had been graded by IDx-DR but was ungraded by all three human graders due to image quality. Eighty-one patients were ungraded by IDx-DR but graded by at least one human grader; nonetheless, there were no patients with full human grading for whom IDx-DR failed to reach a decision. The decisions surrounding whether quality of images was sufficient for grading were at the discretion of the ophthalmologists with no external standard imposed.

Distribution of DR in the studied population, based on human grading, after the process of adjudication, is presented in Table 1. About 27% of patients had a final grade of DR-positive, out of which the majority had mild DR only. Thirty-six or about 11% of patients had more than mild DR, which would be considered as referable DR.

The three human graders achieved a unanimous agreement in over half of eyes studied (58% of eyes and 56% of photos), as shown in Table 2. Furthermore, an agreement of 2 out of 3 ophthalmologists allowed a final grade to be reached in 38% of eyes. Overall, a majority decision was reached by the human graders in 96% of eyes (95% of images), leaving only 28 eyes assigned with three, separate, discordant grades that required further adjudication. Agreement levels were almost identical between left and right eyes.

Analyzing 841 images (385 eyes) with full agreement between all three graders virtually all of those results (94%) are from images judged as no DR present. In 5.20% of eyes, the final grade was mild DR. Moderate and severe DR images with full agreement consisted of less than 1.5% of all images with full agreement combined. There were no eyes where all graders unanimously designated a severe proliferative DR diagnosis. Counts of full-grader agreement on a patient basis, defined as the same final per-patient grade being unanimous between the three graders (as computed from the individual per-eye grades), are shown in Table 3.Unanimous decision of the three human graders was reached mainly in patients with no DR as indicated in Table 4. Only 2 of the 36 patients with a final grade of more than mild DR were designated so unanimously.

The final DR grades of eyes where a majority decision could be reached, that is, in instances where two out of three graders agreed on a particular grade, is shown are Table 5. Compared with eyes with full agreement, there are a lot more DR-positive results, particularly mild and moderate DR. There were 3 eyes identified with a majority decision of proliferative DR, as well as 41 other eyes with more than mild DR.

Human graders designated a single DR grading per eye, in contrast to IDx-DR, which outputs a single grade per patient without distinguishing individual eyes. Additionally, IDX-DR displays results on a 4-point scale instead of the 5-point ICDR scale. In order to contrast the human graders with IDX-DR, the top-end of the DR severity scale was compressed to match the IDX-DR output. This way severe proliferative (grade 5 out of 5) and severe non-proliferative (grade 4 out of 5) DR were joined to form the top end of the 4-point scale—“sight threatening DR”. To form a single per-patient grade from two per-eye grades, the higher result out of both eyes was chosen. For example, if the grade for the right eye is 4 and for the left eye is 2, an overall grade for the patient of 4 was chosen.

Considering the DR software as a fourth grader, almost one-third of patients had full agreement between four graders, as shown in Table 6. A breakdown of these patients is shown in Table 7. For a further 99 patients, a clear majority decision of three out of four graders is evident with IDx-DR being in majority in 25 patients and going against the majority decision of 3 ophthalmologists in 64 patients. Out of 11 patients with 3 different overall human grades, 4 patients had a 4th discordant grade assigned by IDx-DR, meaning that between the human graders and IDx-DR every possible grade from no DR to sight-threatening DR was assigned. These patients are discussed in more detail later on. For a further 7 patients, IDx-DR sided with one of the human graders.

Grader reliability was calculated using Cohen’s kappa statistic. Graders’ reliability scores, measured against the final grading for each eye, were 0.52, 0.78, and 0.61, respectively, for graders 1, 2, and 3. We also analyzed grader agreement by calculating Cohen’s kappa for each pair of graders and averaging the results for each grader, with the highest average kappa score achieved by grader 2 (0.40) and lowest by grader 1 (0.31).

After reviewing the preliminary results, it was clear that we have a decently high grader variance, with a significant proportion of disagreements between graders. We decided to perform a post hoc analysis of the results in a simpler referable DR (rDR) or no referable DR classification. We defined referable DR as more than mild DR (levels moderate DR and above, same as the cutoff set by IDx-DR) and analyzed the same data by collapsing the DR scale into two results rDR or no rDR. The difference between referable and non-referable DR is the most important and crucial step in DR severity for a screening program as it determines further recommendations for each patient. As this is a binary classification, there will always be a majority agreement between 3 graders and therefore using such system adjudication would not be essential for discerning disagreements. We therefore did not include the adjudication in the post hoc analysis and used the original grades assigned.

Using a binary classification of no rDR or rDR there was full agreement of 3 retina specialists regarding 588 eyes and a majority decision of 2 graders in 81 eyes. This translated into full grader agreement on a patient level in 309 patients and majority decision (2:1) for 26 patients. For the 26 patients with a partial grader agreement, IDx-DR sided with the majority of graders in 12 cases and against the majority in 14.

Furthermore, on a patient level, there were disagreements in patients with full grader agreement and IDx-DR in 85 cases. Out of those, IDx-DR suggested a patient is referable in 79 cases against the graders’ result, showing a general tendency to grade higher and safer than the retina specialists in this study.

## 4. Discussion

Interpretation of many ophthalmic examinations, including ophthalmoscopy and fundus image assessment for DR, is inherently subjective. Variability in assessing ophthalmological examinations for DR is not a recent idea. In 1993, Pugh et al. published their comparison of performance of ophthalmologists in detecting and staging DR using direct ophthalmoscopy, a single non-mydriatic fundus photography or three mydriatic fundus images. Their performance was compared with a stereoscopic seven-field study graded by an external reading center [8]. Somewhat surprisingly, the ophthalmologists performed better when reading mydriatic 45-degree fundus photography than when using direct ophthalmoscopy, with the authors concluding mydriatic photography as non-inferior to ophthalmoscopy [8]. DY Lin et al. performed a similar study comparing ophthalmologists’ performance when using direct ophthalmoscopy with a single monochromatic digital image and seven standard stereoscopic 35 mm film images [9]. The results showed excellent agreement between the results from a single digital image compared against the seven-field photography, but poor agreement when ophthalmoscopy was used [9]. Difficulties and subjectivity of interpretation is not limited to DR. AP Lin et al. evaluated interobserver agreement when assessing visual field results for glaucomatous changes [10]. General ophthalmologists and glaucoma specialists evaluated visual fields with different presentation formats (HVF, STATPAC2, and PROGRESSOR). In general, glaucoma specialists had higher agreement scores and analyzed the results faster. General ophthalmologists had median unweighted kappa scores of 0.35–0.43, while glaucoma specialists achieved median kappa scores of 0.43–0.60, depending on the protocol used [10].

Krause et al. examined the variability in grading and its effects on building deep learning models for the detection of DR, with a focus on resolving disagreements between graders [1]. Resolution of a final grade by both a majority decision and an adjudication process for any disagreement (not only full disagreement) between three retina specialists was performed. The authors found that most adjudicated grades remained within 1 degree of severity of the majority decision. However, where adjudication resulted in new grades, these tended to be higher severity grades. They also found majority decision to have a higher sensitivity than any single grader [1].

In the aforementioned study by Krause et al., the errors in non-adjudicated, majority decision grades stemmed mostly from missed MA, misinterpreting artifacts, and disagreements regarding whether a lesion was an MA or a small hemorrhage. In our study four patients deserve special attention in terms of grader disagreement. These four patients had an initial final DR grading that was different for every grader; that is, each of the three retina specialists and IDx-DR designated a different DR severity from no DR to severe DR.

**Figure 2:**
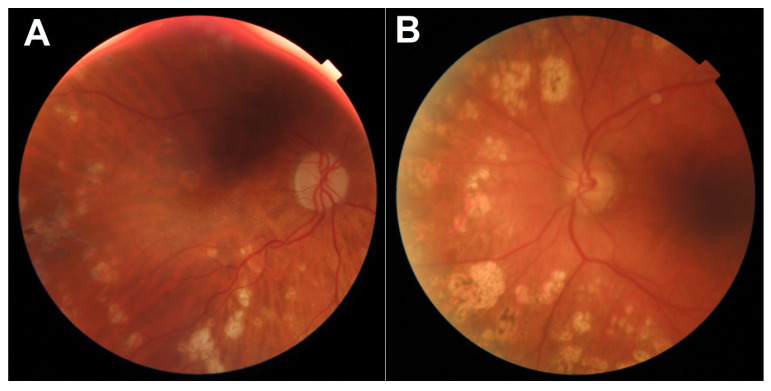
Fundus image of patients (**A**,**B**), showing multiple retinal photocoagulation scars.

**Figure 3:**
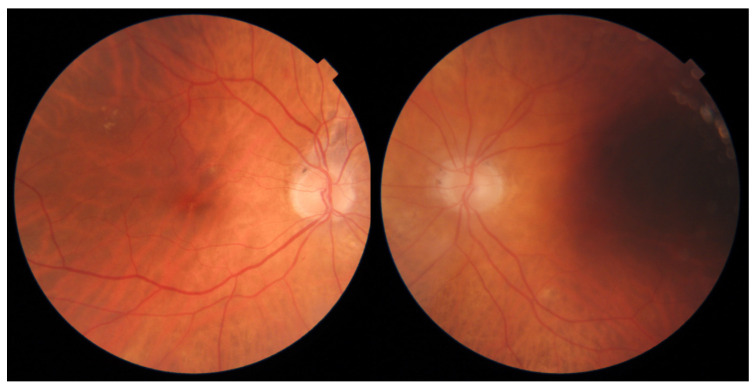
Fundus images of patient C, showing peripapillary pigment changes, hard exudates in peripheral macula, and more subtle exudates near the fovea in the **right** eye, and low quality of images with a large shadow in the **left** eye.

**Figure 4:**
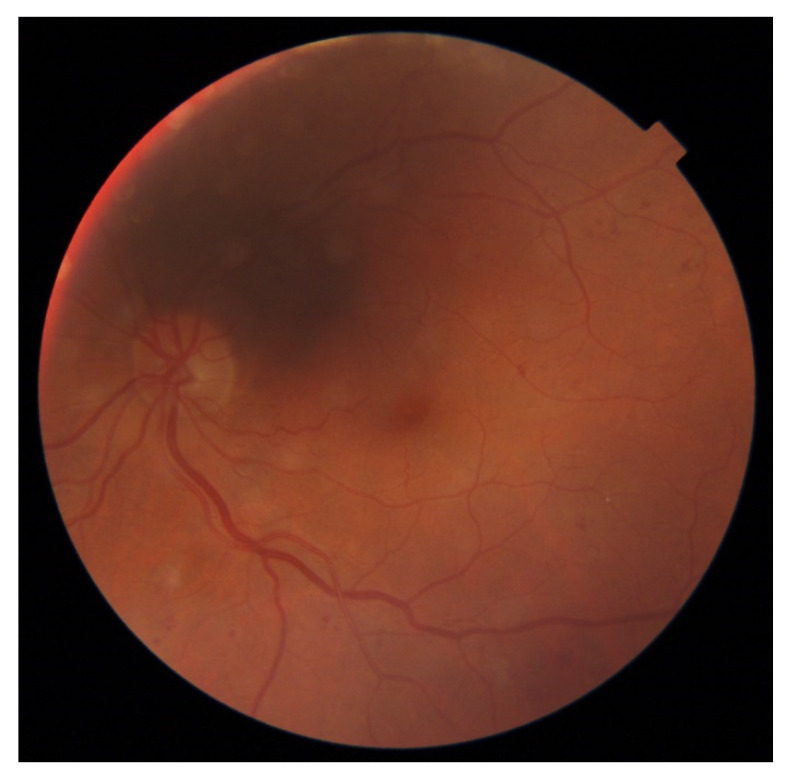
Fundus image of patient D, showing multiple small hemorrhages, microaneurysms, and multiple image artifacts.

These patients subsequently underwent an adjudication process, the results of which are shown in Table 7. Two of the disagreements stemmed from patients with scars from previous pan-retinal photocoagulation, as shown in Figure 2. These patients are not the target population for routine DR screening and instead should be routinely followed up in ophthalmology clinics. In general, such patients are regarded as having proliferative DR regardless of whether the proliferations are currently active and therefore should be followed closely in dedicated specialty clinics, lending credence to the severe DR diagnosis designated by IDx-DR. For those cases, the difference in grading stemmed mainly from the procedural uncertainty of grading such patients, rather than missed or misinterpreted lesions. For the other two patients, shown in Figure 3 and Figure 4, a root cause of some graders’ results is difficult to adequately explain. In the face of multiple and repeatable lesions, we consider a designated grading level of no DR as a manifestation of the inherent variability and fallibility of human grading.

One of the difficulties regarding the detection of small and subtle retinal changes stems from image artifacts. These are often a result of dust specs or other consistent camera lens opacities causing repeatable problems with interpretation. This is partly alleviated when grading multiple fields from a single eye, as in this study; artifacts may be repeated between images.

For each patient grade designated by IDx-DR, the software takes a macula- and a disc-centered image from each eye, four images total. The software is equipped with an image-quality assessment algorithm and prompts the user to retake any images deemed to be of insufficient quality. Only once four suitable images are selected IDx-DR will display a patient DR grade. As a result of the quality-assurance process some patients will have more than two images taken per-eye, until images of suitable quality are taken, or a patient encounter is marked as insufficient quality and sent for a human grader assessment.

There have been numerous studies looking into the performance of AI DR software against human graders of different specialty and training levels, and AI DR algorithms show significant performance differences [11,12]. Recently Sedova and colleagues compared the performance of IDx-DR with seven-field ETDRS protocol fundus images and ultra-wide-field images graded by two retinal specialists [13]. They found IDx-DR results to have a 66.6% and 66.7% agreement with seven-field and ultra-wide-field examinations, respectively, with respective kappa scores of 0.40 and 0.38 [13]. Of note, in the current study, both AI and human graders had access to the same information (only standard color macula- and disc-centered fundus images), whereas Sedova et al. compared IDx-DR output to graders using more comprehensive imaging studies.

There are several difficulties in assessing and comparing DR grader variability and reliability, and many more when attempting to contrast human graders with automatic grading systems.

The first issue regarding grading is selection of a grading scale and protocol. Although the ETDRS grading scheme is the probably the most validated and extensive grading protocol that is widely accepted, it was designed for a seven-field fundus assessment which is not feasible for most larger-scale studies. Additionally, the ETDRS grading scheme is extensive and requires comparison with standardized images, which overall makes it a very time- and labor-intensive process for the graders. Most deep learning DR detection systems do not specifically state adherence to a certain grading system, and studies into AI DR detection systems vary in the grading scheme employed. Reliability and accuracy of human and machine graders is likely to vary depending on the grading scheme used as shown by Abramoff et al. in their study comparing IDx-DR performance against ICDR and EURODIAB DR grading [14].

A second issue influencing final reliability measures and complicating direct comparisons between studies is the grading level. A DR grade can be designated to an individual image, to an eye from multiple fundus images of the same eye, or to a patient from multiple images from one or both eyes. Studies focusing on retrospective image databases mostly chose to focus on a per-image grading as that is the data available to them [1,15,16]. A “per-image” approach is probably the simplest and most easily compared between studies and algorithms; however, it is the furthest from a real clinical screening scenario where image quality and region of the fundus that is visualized may vary. Additionally, multiple images per eye are typically taken without impacting the workload and flow of a screening program significantly. In comparison with a per-image grade, an overall per-eye grade is more comparable with a real clinical screening scenario where a decision regarding patient referral needs to be made. Both ICDR and ETDRS grading scales differentiate between the number of features or quadrants with given DR features, which is rarely feasible in grading a single fundus image. A third method designates a single overall grade per patient, which from screening and epidemiological perspectives provides actionable results. However, from a scientific perspective, it is hard to assess and particularly difficult to resolve and make sense of differences between individual graders or algorithms. With a single patient grade, back-tracing the origins of discordant grades and disagreements is difficult, because they could stem from any image or eye. On the other hand, a per-patient grade is the closest comparison to a traditional direct visualization by an ophthalmologist screening method and directly represents a final referral decision for a given patient. There are examples of all three grading levels employed within different automatic DR grading systems [11].

The different grading levels complicate attempts at direct comparison of systems and of DR grading systems with human graders, as they necessitate the need for additional data manipulation. In this study, we decided on a per-eye classification for the human graders, as there were a variable number of images per-eye and per patient. This necessitated a conversion from per-eye grades to per-patient grades, both to assess the prevalence of DR stages and to compare results with those from IDx-DR. Each individual grader’s higher per-eye grade for a given patient was designated as the overall patient-level grade and these were later compared for a per-patient grading decision, therefore simulating a real-life screening scenario.

Previous studies reported a wide range of interobserver and grader reliability, ranging from 0.22 to 0.91 [1,6,17,18,19,20]. The individual observer reliability kappa scores of 0.52, 0.78, and 0.61 are within the range of values previously reported in the literature, though a closer analysis of previous studies shows that major differences in study design obscure meaningful ways of quantitative comparison. As mentioned before, some studies use different grading levels than the per-eye grading employed by this study; additionally, previous studies differ in calculating weighed or unweighted kappa scores. Some studies compared grader performance against reading center results as the gold standard. An overview of results from studies evaluating human grading of DR is shown in Table 8.

## 5. Conclusions

Human grading of DR severity from fundus images is hindered by significant inter-grader variability, even among specialists diagnosing and treating DR and other retinopathies on a day-to-day basis. The everyday ophthalmic practice does not necessarily translate into consistent and adequate image-based grading. This may be partly alleviated by purpose-built training, quality-assurance steps, and using multiple graders and an adjudication process for more challenging cases.

In the face of the variability of human grading, a fair comparison of human and software graders is difficult without establishing an external gold standard. From a scientific standpoint, even when such a standard is available, the translation of human grader results to be comparable with IDx-DR results required multiple steps of data transformation. This is based on different severity level scales used and displaying per-eye or per-patient results. In consequence, where disagreements arise, their root is often difficult to establish.

## Figures and Tables

**Table 1:** Distribution of diabetic retinopathy (DR) grades per patient after final adjudication, based on human grading.

DR Grade (4-Point Scale)	Number of Patients	Percentage of Patients
0	245	73.13%
1	54	16.12%
2	29	8.66%
3	7	2.09%

**Table 2:** The number of assessments of fundus images by proportion of agreement (5-point scale).

Proportion	Number of Images	Percentage of All Images	Eyes	Percentage of All Eyes Studied
1:1:1 (both eyes)	72	5%	28	4%
1:1:1 (left eye)	41	1%	15	2%
1:1:1 (right eye)	31	1%	13	2%
2:1 (both eyes)	588	39%	256	38%
2:1 (left eye)	294	9%	129	19%
2:1 (right eye)	294	8%	127	19%
3:0 (both eyes)	841	56%	385	58%
3:0 (left eye)	406	13%	190	28%
3:0 (right eye)	435	13%	195	29%

**Table 3:** Counts of images in each DR severity level with full grader agreement.

DR Grade (5-Point Scale)	Number of Images	Percentage of Images	Number of Eyes	Percentage of All Eyes
0	789	9.00	361	93.77
1	40	4.76	20	5.20
2	6	0.71	3	0.78
3	6	0.71	1	0.25
4	0	0.00	0	0.00

**Table 4:** Count of patients in each DR stage with full grader agreement.

DR Grade (4-Point Scale)	Number of Patients	Percentage
0	108	96.42
1	2	1.79
2	0	0.00
3	2	1.79

**Table 5:** Counts of images and eyes in each DR severity level with majority grader agreement (2 out of 3 graders agree on severity level).

DR Grade (5-Point Scale)	Number of Images	Percentage of Images	Number of Eyes	Percentage of All Eyes
0	331	49	149	58.20
1	142	21	62	24.22
2	94	14	37	14.45
3	15	2	5	1.95
4	6	1	3	1.17

**Table 6:** The number of the highest assessments assigned to patients.

Proportion	Number of Patients	Percentage
1:1:1:1	4	1.09
2:1:1	80	21.80
2:1:1 (the assessment assigned by the IDx-DR is in majority graders’ assigned scores)	7	1.91
2:2	40	10.90
3:1	99	26.97
3:1 (the assessment assigned by the IDx-DR is in majority human graders’ assigned scores)	25	6.81
4:0	112	30.52

**Table 7:** Breakdown of results for 4 patients with total disagreement between graders and IDx-DR. Letters A to D represent individual patients.

Patient	Grader 1		Grader 2		Grader 3		Adjudicated Grades for OD/OS	IDx-DR Patient Level Grade
A	0	0	1	1	2	2	2/2	3
B	0	0	1	1	2	2	1/1	3
C	2	2	0	0	1	0	1/1	3
D	0	0	1	1	2	2	1/1	3

**Table 8:** Summary of studies reporting grader reliability statistics in grading for diabetic retinopathy.

Study	Grading Level	Sample Size	Grading Details	Comparison	Kappa Scores
**Scott et al.** [20]	Per eye	118 eyes	Four levels of grading based on ophthalmoscopy	Reading centre grading based on 7-field fundus photography	Unweighted kappa 0.55
**Raumviboonsuk et al.** [19]	Per image	400 images	Single-field digital fundus images read by ophthalmologists, retinal specialists, and non-physician staff	Interobserver variability	Overall, 0.34 for retinopathy severity, 0.28 for referral cases; for retinal specialists 0.58, for retinopathy severity, and 0.63 for referrals
**Gangaputra et al.** [18]	Per eye	6902 and 3638 eyes	Eyes taken from two large studies—ACCORD and FIND, 5-level DR severity scale	Reading center	0.42 and 0.65 for FIND and ACCORD, respectively
**ETDRS research group** [6]	Per eye	7402 eyes	Detection of specific features: group 1–retinal haemorrhages, microaneurysms, hard exudates, new vessels, fibrous proliferations, and macular oedema; group 2—soft exudates, intraretinal microvascular abnormalities, venous beading; 7-field fundus images	Interobserver variability	Weighted-kappa—0.61–0.80 for group 1 features; 0.41–0.60 for group 2
**Krause et al.** [1]	Per image		Retinal specialists, ophthalmologists, and a deep-learning-based algorithm, initially graded at a 5-point scale, calculated for various DR severity cutoffs	Adjudicated consensus of retinal specialists	Quadratic-weighted kappa; retinal specialists—0.82–0.91; ophthalmologists—0.80–0.84; 0.84 for the algorithm
**Wang et al.** [17]	Per eye	1589 images	Detection of specific features by individual graders, later computed into severity levels, comparison of different annotation protocols, and methods	Pair-wise intergrader variability calculated for each grader pair for feature and severity detection	Quadratic-weighted kappa; 0.217–0.863 for detection of specific DR features, 0.430–0.914 for DR severity
